# Tolerability of repetitive dihydroergotamine infusions paired with an adjustment in preventive treatment strategy in chronic headache disorders in children and youth

**DOI:** 10.1186/s10194-025-02035-x

**Published:** 2025-04-30

**Authors:** Sara Pavitt, Cynthia Morris, Lauren Shin, Andrew Jones, Beata Vayngortin, Natalia Zorrilla, Chengshi Jin, Isabel Allen, Amy A. Gelfand

**Affiliations:** 1 Department of Neurology, University of Texas at Austin Dell Medical School, 4910 Mueller Blvd, Suite 300 Austin TX, Austin, TX 78723 USA; 2 Department of Neurology, Saint Louis University, 1225 S Grand, Blvd. St Louis, Saint Louis, MO USA; 3 Child & Adolescent Headache Program, University of California San Francisco, 1825 4 th Street, 5 th floor 5 A San Francisco, San Francisco, CA 94143 USA; 4 Department of Internal Medicine, Kaiser Permanente San Francisco, 2425 Geary Blvd. San Francisco, San Francisco, CA 94115 USA; 5 Department of Epidemiology and Biostatistics, University of California San Francisco, 1825 4 th Street, 5 th floor 5 A San Francisco, San Francisco, CA 94143 USA

**Keywords:** Pediatric headache, Dihydroergotamine, Chronic migraine

## Abstract

**Background:**

In adults, intravenous (IV) dihydroergotamine (DHE) has been shown to be effective at improving medium term outcomes in patients with chronic headache disorders. The IV formulation is utilized given its superior bioavailability. We aim to assess the safety and effectiveness of repetitive IV DHE infusions paired with an adjustment of a preventive treatment strategy within children and youth with chronic headache disorders.

**Methods:**

A retrospective chart review was conducted of children and youth diagnosed with a chronic headache disorder who were admitted for DHE from January 2014 – October 2020. Patients completed a 5-day, standardized protocol. A new preventive was started one week after discharge. Data were collected from pre- and post-admission clinic notes. Safety and tolerability were assessed. Results were evaluated using descriptive statistics and compared with paired *t*-tests.

**Results:**

One hundred and eighty-seven patients were included for review. Sixty-eight percent (127) had chronic migraine (CM), 20% (37) new daily persistent headache (NDPH) and 12% (23) persistent headache attributed to head trauma (PHHT). The median (range) age was 16 years (7–21), and median (range) number of previous preventive trials was 4 (0–21). At follow-up, patients with CM had a significant decrease in headache days per month from 28.6 to 26.3 days (95% CI -4.1 to -1.3) *p* < 0.001, baseline headache intensity decreased from 5.9/10 to 5.3/10 (95% CI -1.3 to -0.1) *p* = 0.006, number of severe headache days per month decreased from 11.5 to 7.9 days (95% CI -6.5 to -2.3), *p* < 0.001, and monthly days of acute medication use from 12.1 to 9.8 days (95% CI -4.5 to -0.7) *p* = 0.002. In patients with NDPH there were significant decreases in baseline headache intensity from 6.4/10 to 5.3/10 (95% CI -1.7 to -0.3) *p* = 0.005 and monthly days of acute medication usage from 9.2 days to 5.9 days (95% CI -7.8 to -0.1) *p* = 0.043. Patients with PHHT had a significant decrease in headache days per month from 29 to 24 days (95% CI -9.4 to -0.7) *p* = 0.031. The most common side effects were nausea (85%) and mild leg cramping (60%).

**Conclusion:**

Repetitive DHE infusions followed by preventive treatment adjustment was well tolerated and significantly reduced headache frequency, baseline intensity, number of severe days and/or acute medication usage in children and youth with refractory headache disorders.

**Supplementary Information:**

The online version contains supplementary material available at 10.1186/s10194-025-02035-x.

## Introduction

Chronic headache disorders are common and can lead to significant disability, interfering with all aspects of a child’s life [1, 2]. Furthermore, a dose effect occurs, in which higher headache frequency and severity is directly related to increased disability [3, 4]. Chronic migraine (CM), new daily persistent headache (NDPH), and persistent headache attributed to head trauma (PHHT) are all high frequency headache disorders that can lead to significant disability and be refractory to treatment [5]. Furthermore, patients with refractory headache disorders are often excluded from clinical trials [6] leading to scarcity of evidence to direct management.

One treatment strategy for refractory headache disorders is intravenous (IV) medication in an inpatient setting. Dihydroergotamine (DHE) is an ergot alkaloid synthesized from ergotamine tartrate. Its therapeutic mechanism of action is hypothesized to be from binding serotonin (5-hydroxytryptamine) receptors type 1D and 1B, binding dopamine D2L and D3 receptors, and activating alpha 2- adrenoceptors causing cranial vasoconstriction and inhibition of release of inflammatory peptides in the trigeminovascular complex including calcitonin gene-related peptide (CGRP) [7, 8]. IV administration of DHE is often used as acute treatment for migraine and other headache disorders, but it has also been shown to be effective as a preventive with improvement in medium-term (6 weeks) outcomes in adults with chronic headache disorders [9, 10]. There is evidence for using DHE for acute and refractory headaches in the pediatric population [11–13]. However, limited data have been published for standardized use of DHE as a preventive in youth with chronic headache disorders.

For children and youth with refractory chronic headache disorders, the study site offers elective inpatient admission as part of their preventive treatment. They undergo a standardized treatment protocol in which they receive repetitive DHE infusions paired with a new prophylactic medication or adjustment to their current prophylactic medication 1 week following discharge. The 1 week period allows sufficient time for DHE to metabolize and to differentiate adverse effects of DHE following admission from the adverse effects of the new or adjusted preventive medication. This protocol was developed through consensus of the pediatric headache specialists in the center based on published evidence for the use of IV DHE as a preventive strategy [10, 14]. While there are alternative formulations of DHE, the bioavailability is much less compared to the IV formulation, which has guided its use in this clinical context [15, 16].

We hypothesized that using repetitive DHE paired with an adjustment of preventive treatment strategy would be well tolerated and reduce monthly headache days and/or headache intensity as well as decrease use of acute treatment within 6–8 weeks of discharge.

## Methods

This study was approved by the University of California San Francisco Institutional Review Board as part of a standing approval for chart review research on children and youth with headache disorders (16–18,622). The need for written informed consent was waived.

We conducted a retrospective chart review of all patients diagnosed with chronic headache disorders who were admitted for IV DHE for preventive treatment at the University of California—San Francisco from January 2014 to October 2020. This is the primary analysis of these data, except for safety data around deep vein thrombosis (DVT) has been previously reported for some of these patients [17]. Prior to admission, all patients were evaluated by a pediatric headache specialist and diagnoses were based on International Classification of Headache Disorders (ICHD) 2nd or 3rd edition criteria, depending on the year [18, 19]. Patients ≤ 21 years who met criteria for CM, NDPH, or PHHT were included for review. Patients were excluded if they were older than 21, received DHE for an alternative diagnosis or did not have a follow up appointment documented in the electronic medical record.

**Fig. 1:**
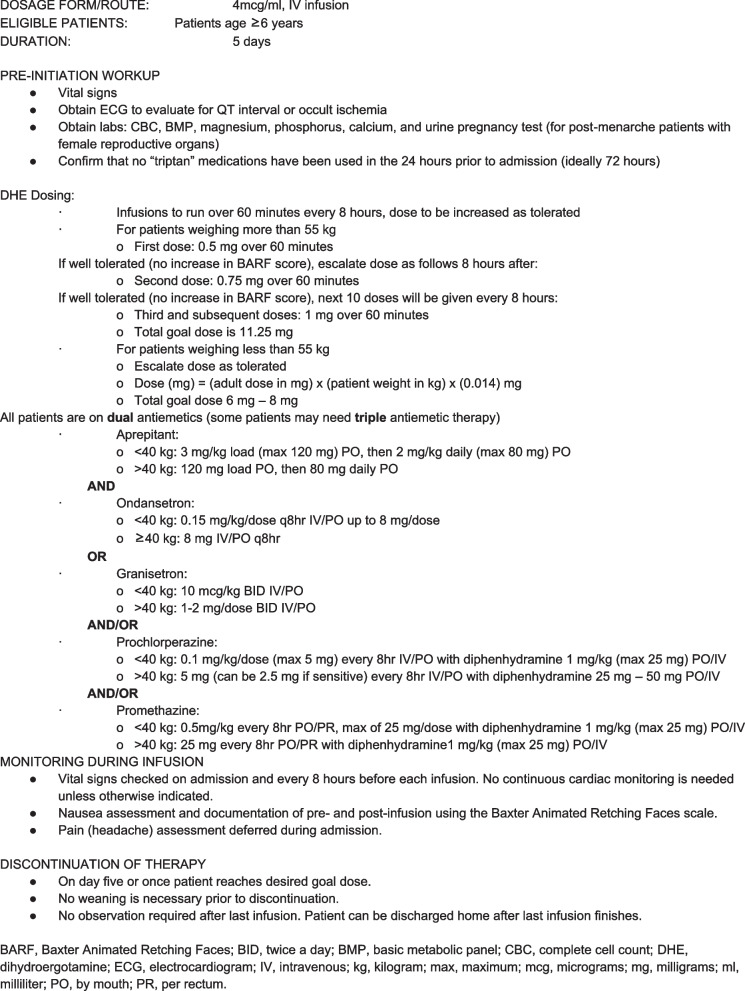
An adapted version of the University of California San Francisco treatment guideline for the use of intravenous dihydroergotamine in children and youth with headache disorders

All patients underwent the same 5-day standardized DHE protocol, modified from Nagy et al. [10] DHE dosing was weight adjusted for patients under 55 kg (Fig. 1).

The protocol consists of 12 total weight-based doses of DHE. Two lower “test doses’ are given to ensure adequate nausea control prior to the remaining 10 full doses. Before starting the infusions, patients had a baseline electrocardiogram, complete blood count, basic metabolic panel, magnesium, phosphorus, calcium and urine pregnancy test (for post-menarche patients with female reproductive organs). Patients could not proceed with the infusion if they had taken a triptan within 24 h. To assess for possible cardiac risk, and because some of the anti-nausea medications given can prolong the QT interval, a baseline electrocardiogram is obtained prior to starting DHE. Vital signs are collected before each infusion, but continuous cardiac monitoring is not needed unless the clinical scenario requires it.

**Fig. 2:**
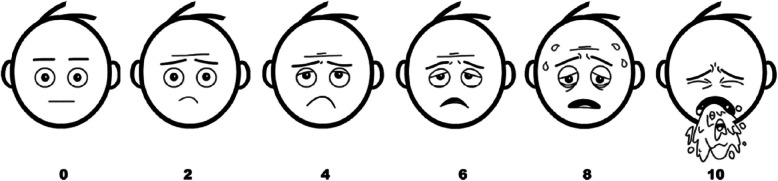
Baxter Animated Retching Faces (BARF) Nausea Assessment Scale [20]. Reprinted with permission

Nausea is a known common adverse effect of DHE and nausea control is a positive predictor of overall response to DHE [14]. Therefore, changes in nausea levels were closely followed during the admission for all patients receiving DHE and all patients were started on dual antiemetic therapy (Fig. 1). The Baxter Animated Retching Faces (BARF) score was taken before and after each infusion (Fig. 2) [20] to assess severity of nausea.

If the BARF score exceeded 4 or increased by ≥ 2 points from baseline, antiemetic therapy was escalated.

During admission, patients continued their home headache preventive medications, but acute medications were held. At our program, we targeted medium term (6 to 8 week) headache benefits; therefore, patients were not asked to rate their pain scores during admission. One week after discharge, patients were generally started on a new preventive strategy.

Four authors completed chart review (S.P., C.M., B.V., and N.Z.). Notes were reviewed for the appointment immediately prior to admission, during admission, and at the subsequent follow up visit (6–8 weeks post admission). A standardized abstraction form was used between all reviewers, and all providers in the pediatric headache program used the same note template. Data were entered into a secure Research Electronic Data Capture (REDCap) database [21]. Patient demographics and clinical data were collected, which included headache frequency (days per month), severe headache frequency (days per month), baseline pain intensity (on a scale of 0–10), severe headache intensity (on a scale of 0–10) and frequency of acute medication usage (discrete medication usage days per week). If a range was given (i.e., headache pain 3–4/10) the higher number was used. Patients were asked to recall variables over the time since their previous clinic appointment. PedMIDAS, a validated six question instrument to measure headache-related disability in children and adolescents, was collected as a measurement of pre-admission disability [22]. It was not collected post-admission as the follow-up appointment fell within the same 3-month time frame. The vast majority of patients evaluated in this clinical practice are children and adolescents, therefore, the PedMIDAS tool is universally used for all patients regardless of age. Variables were excluded if they had greater than 20% missing data. Only first-time DHE admissions were included in the effectiveness analyses to mitigate selection bias. Safety and tolerability were assessed with all DHE admissions (first time and repeat). Information was collected through the patient’s subjective narrative, laboratory findings and order review during admission and the post-admission follow-up.

### Statistical analysis plan

Initially, descriptive summaries were calculated for all participants. Continuous characteristics of the study cohort were summarized using mean, standard deviation, minimum, median, and maximum. Categorical variables were summarized using frequencies and percentages.

Change in monthly headache days, headache intensity, and frequency of acute medication usage based on headache diagnosis (CM, NDPH, and PHHT) pre- vs. post- admission were summarized by mean and 95% confidence intervals (95% CI) and pre- vs. post-admission differences in median were assessed using Wilcoxon signed rank tests. Data were rounded to the degree of precision with which they were measured to avoid false precision.

Seven variables were chosen a priori to assess their possible association with change in monthly headache days. These variables included: change in nausea score, number of previous preventive trials, headache diagnosis, presence of mild leg cramping, presence of severe leg cramping, and presence of cognitive behavioral therapy prior to admission, and presence of comorbid mood disorder. The association between change in monthly headache days and these variables pre- vs. post- admission were assessed using univariable linear regression models and then multivariate regression modeling.

For all analyses, *p* < 0.05 in a two-tailed test was considered the criterion for statistical significance. Statistical analyses were performed using SAS software version 9.4 (SAS Institute, Cary, NC).

## Results

### Demographics

Two hundred and forty-one patients were identified for review. Fifty-four patients were removed due to exclusion criteria. Therefore, 187 unique patient records were included in the analysis for first-time DHE admission. An additional 176 hospital encounters for repeat DHE admissions were included in review of safety data.

For first-time DHE admissions, headache diagnosis was CM (*n* = 127), NDPH (*n* = 37) or PHHT (*n* = 23). Most patients were adolescents (median age 16 years, range 7–21) and female (74.3%). Nineteen patients were between 18–21 years old. Median time to follow up was 7 weeks, (IQR 5–9). Demographics are shown in Table 1.

**Table 1:** Demographics for patients with chronic headache disorders

	**Chronic Migraine** **(*n* = 127)**	**New Daily Persistent Headache** **(*n* = 37)**	**Persistent Headache Attributed to Head Trauma** **(*n* = 23)**
Median age (min–max), years	16 (7–21)	16 (9–21)	17 (13–21)
Sex assigned at birth, n (%)	Female, 94 (74.0) Male, 31 (24.4)	Female, 25 (67.6) Male, 12 (32.4)	Female, 20 (87.0) Male, 3 (13.0)
Comorbid mood disorder(s), n(%)	53 (41.7%)	12 (32.4%)	13 (56.5%)
Diagnosis of other pain disorder(s), n (%)	8 (6.3%)	1 (2.7%)	1 (4.3%)
Presence of continuous headache at time of admission, n (%)	71 (55.9%)	37 (100%)	15 (65.2%)
Median number of previous preventive trials (min–max)	4 (0–10)	3 (1–10)	4 (0–21)
Previous trial of CGRP mAb prior to admission, n (%)	7 (5.5%)	1 (2.7%)	1 (4.3%)
CBT prior to admission, n (%)	35 (27.6%)	12 (32.4%)	9 (39.1%)
Pre-admission PedMIDAS score, Mean (SD)/Median (range)	141 (82.6)/130 (4–270) (*n* = 57)	144 (78.7)/134 (4–246) (*n* = 15)	168 (88.5)/149 (40–270) (*n* = 9)

*Abbreviations: CBT* cognitive behavioral therapy, *CGPR mAb* calcitonin gene-related peptide monoclonal antibody, *PedMIDAS* Pediatric Migraine Disability Assessment, *SD* standard deviation

Of the patients with CM, 56% (71/127) had daily continuous headache, and of the patients with PHHT, 65% (15/23) had daily continuous headache. All patients with NDPH had daily continuous headache.

There were comorbid mood disorders in 41.7% of patients with CM, 32.4% with NDPH, and 56.5% with PHHT. Most patients did not have other chronic pain disorders. All three groups had severe disability as evidenced by high median (range) PedMIDAS scores 130 (4–270), 134 (4–246), and 149 (40–270) in CM, NDPH, and PHHT groups respectively. The median (range) number of preventive trials prior to DHE administration was 4 (0–21). The majority had not tried CGRP targeted therapy prior to the admission. Of note, CGRP pathway monoclonal antibodies and antagonists were yet to be widely used during most of the study period.

Following admission, 76.4% (CM), 78.4% (NDPH), and 87% (PHHT) of patients had new preventive treatment initiated. These included: propranolol, topiramate, erenumab, candesartan, memantine, venlafaxine, amitriptyline, nortriptyline, coenzyme Q10, riboflavin, melatonin, and neuromodulation devices such as transcutaneous supraorbital nerve stimulation. Another 19.7% (CM), 16.3% (NDPH), and 4.3% (PHHT) of patients had adjustment to their pre-admission preventive treatment following the admission (for example, an increase in dosage of a medication they were already taking).

### Outcomes

#### Chronic Migraine

**Fig. 3:**
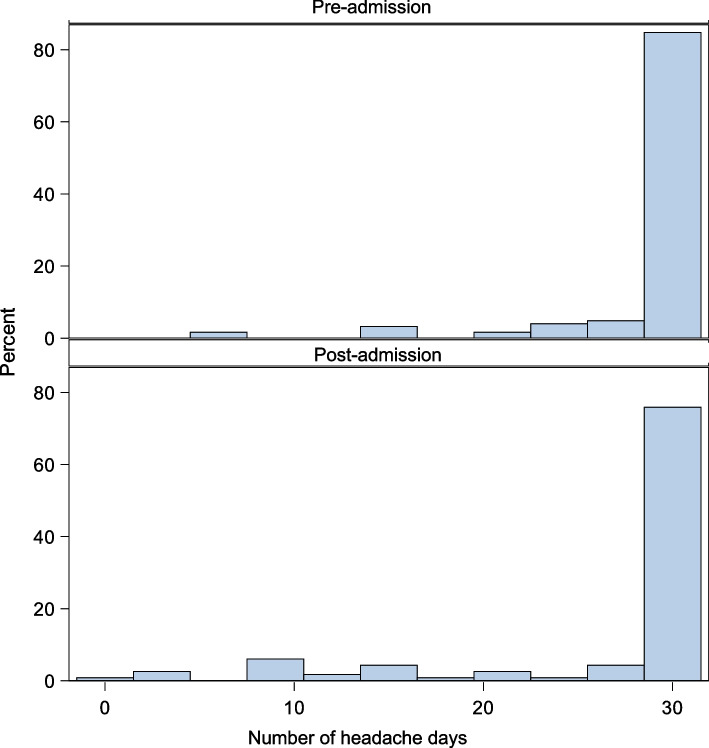
Histogram depicting pre-admission and post admission monthly headache days for patients with chronic migraine

Mean headache days per month prior to DHE infusion was 29 and post-DHE was 26 days per month. The mean reduction in headache days at 6-to- 8-week follow-up was 3 days (95% CI − 4.1 to − 1.3, P < 0.001). Twelve patients (9.5%) had a greater than 50% reduction in monthly headache days. Mean baseline pain intensity was 6/10 prior to admission and decreased to 5/10 post-admission, with a mean difference at follow-up of − 0.70 (95% CI for the difference: − 1.3 to − 0.14, *P* = 0.006). Mean severe headache days per month pre-admission was 12 days and post-admission was 8 days, with a mean difference of − 4.4 (95% CI − 6.5 to − 2.3, *P* < 0.001). Acute medication usage decreased from 12 days per month before admission to 10 days per month after admission, with a mean difference of − 2.56 (95% CI − 4.48 to − 0.66, *P* = 0.002) (Table 2, Fig. 3, Supplementary Fig. 1).

**Table 2:** Monthly headache frequency, intensity, and acute medication frequency before and after admission in patients with chronic migraine

Clinical Characteristics	Before Admission	Post Admission Follow up Appointment	Mean Difference (95% CI)
Mean headache frequency, days per month (SD)	29 (SD 4.2) (*n* = 125)	26 (SD 7.7) *(n* = 116)	− 2.7 (− 4.1 to − 1.3, *P* < 0.001)
Mean baseline pain intensity on a scale 0–10 (SD)	6 (SD 2.1) (*n* = 69)	5 (SD 2.2) (*n* = 60)	− 0.7 (− 1.3 to − 0.1, *P* = 0.006)
Mean severe headache frequency, days per month (SD)	12 (SD 6.1) (*n* = 62)	8 (SD 5.5) (*n* = 48)	− 4.4 (− 6.5 to − 2.3, *P* < 0.001)
Mean acute medication use, days per month (SD)	12 (SD 8.7) (*n* = 104)	10 (SD 8.0) (*n* = 87)	− 2.5 (− 4.5 to − 0.7, *P* = 0.002)

Bolded values are significant with *P* < 0.05

##### NDPH

**Fig. 4:**
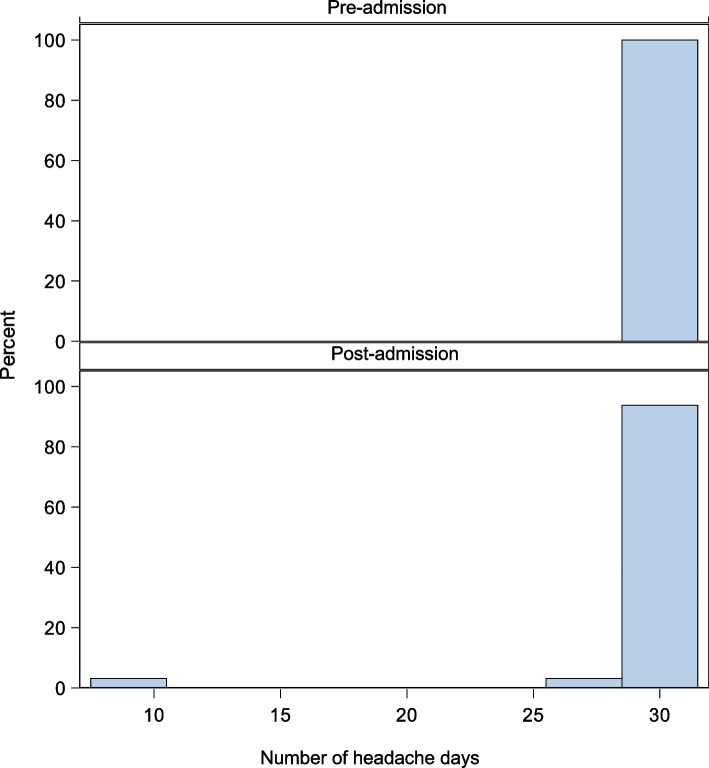
Histogram depicting pre-admission and post admission monthly headache days for patients with new daily persistent headache

Mean baseline pain intensity was 6/10 prior to admission and decreased to 5/10 post-admission with mean difference at follow up of 1.0 (95% CI: − 1.74 to − 0.34, *P* = 0.005). One patient (2.7%) had a greater than 50% reduction in monthly headache days. Acute medication usage for headaches decreased from 9 days per month before admission to 6 days per month after admission, with a mean difference of − 3.9 (95% CI: –7.8 to − 0.1, *P* = 0.043). Change in mean monthly headache days and monthly severe days did show significant difference (30 days vs. 29 days; mean difference − 0.8 days (95% CI, − 2.2 to 0.6, *p* = 0.500)) (Table 3, Fig. 4, Supplementary Fig. 2).

**Table 3:** Monthly headache frequency, intensity, and acute medication frequency before and after admission in patients with new persistent daily headache

Clinical Characteristics	Before Admission	Post Admission Follow up Appointment	Mean Difference (95% CI)
Mean headache frequency, days per month (SD)	30 (SD 0.0) (*n* = 34)	29 (SD 3.9) (*n* = 32)	− 0.8 (− 2.2 to 0.6, *P* = 0.500)
Mean baseline pain intensity on a scale 0–10 (SD)	6 (SD 1.8) (*n* = 25)	5 (SD 2.2) (*n* = 28)	− 1.0 (− 1.7 to − 0.3, *P* = 0.005)
Mean severe headache frequency, days per month	9 (SD 7.1) (*n* = 22)	6 (SD 5.2) (*n* = 16)	− 3.5 (− 7.6 to 0.5, *P* = 0.074)
Mean acute medication use, days per month (SD)	9 (SD 7.1) (*n* = 26)	6 (SD 5.2) (*n* = 28)	− 3.9 (− 7.8 to − 0.1, *P* = 0.043)

Bolded values are significant with *P* < 0.05

*Abbreviation: SD* standard deviation

##### PHHT

**Fig. 5:**
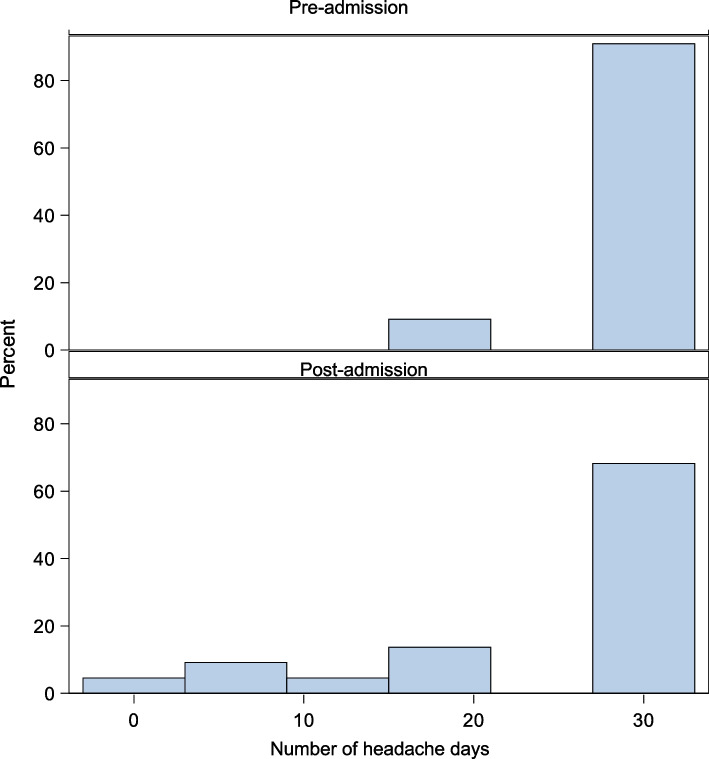
Histogram depicting pre-admission and post admission monthly headache days for patients with persistent post traumatic headache

Mean headache days per month prior to DHE infusion was 29 and at follow up appointment, was 24 days per month. The mean reduction in headache days at 6 to 8 week follow up was − 5.0 days (95% CI: − 9.43 to − 0.7, *P* = 0.031). Four patients (17.4%) had a greater than 50% reduction in monthly headache days. There was no significant change in baseline headache intensity, mean number of severe headache days or acute medication usage (Table 4, Fig. 5, Supplementary Fig. 3).

**Table 4:** Monthly headache frequency, intensity, and acute medication frequency before and after admission in patients with persistent headache attributed to head trauma

Clinical Characteristics	Before Admission	Post Admission Follow up Appointment	Mean Difference (95% CI)
Mean headache frequency, days per month (SD)	29 (SD 2.9) (*n* = 22)	24 (SD 9.8) (*n* = 22)	− 5.0 (− 9.4 to − 0.7, *P* = 0.031)
Mean baseline pain intensity on a scale 0–10 (SD)	4 (SD 2.2) (*n* = 10)	5 (SD 1.5) (*n* = 9)	− 0.0 (− 4.4 to 4.4), *P* = 1.000)
Mean severe headache frequency, days per month	17 (SD 8.2) (*n* = 8)	9 (SD 9.8) (*n* = 10)	− 7.8 (− 16.7 to 1.1, *P* = 0.130)
Mean acute medication use, days per month (SD)	11 (SD 10.6) (*n* = 17)	6 (SD 7.1) (*n* = 14)	− 2.4 (− 7.4 to 2.6, *P* = 0.420)

*Note:* Bolded values are significant with *P* < 0.05

*Abbreviation: SD* standard deviation

More than half, 53.5% (68/127), of patients with CM, 32% (12/37) of patients with NDPH, and 52% (12/23) of patients with PHHT reported improvement in baseline headache frequency, headache intensity, frequency of acute medication use, and/or subjective functional improvement. Of note, two patients with NDPH reported resolution of continuous headache after DHE treatment.

#### Associations with response

In univariate analysis, PHHT had a significant association of benefit (defined as reduction of headache days per month) when compared to patients with NDPH (− 4.2; 95% CI: − 8.3 to − 0.2, *p* = 0.040). However, this was no longer significant after multivariate analysis (− 3.5; 95% CI: − 7.4 to 0.4, p = 0.080). There were no other statistically significant associations for response to therapy (Table 5).

**Table 5:** Univariate linear regression model for predictors of difference in headache days in patients treated with IV dihydroergotamine

Predictor	Beta Coefficient (95% CI)	*P* value
Change in nausea score	− 0.316 (− 1.534 to 0.902)	0.61
Number of previous preventive trials	0.004 (− 4.443 to 4.246)	0.99
Headache diagnosis		
a. NDPH b. CM c. PTTH	a. 0.00 (reference) b. − 1.889 (− 4.808 to 1.030) c. − 4.233 (− 8.274 to − 0.192)	a. - b. 0.20 c. 0.040
Presence of comorbid mood disorder	− 1.441 (− 3.728 to 0.846	0.22
Presence of mild leg cramping	− 0.343 (− 2.664 to 1.958)	0.77
Presence of severe leg cramping	2.318 (− 4.363 to 8.998)	0.49
Cognitive behavioral therapy treatment prior to admission	− 0.073 (- 2.559 to 2.414)	0.95

*Note:* Bolded values are significant with *P* < 0.05

*Abbreviations: CI* confidence interval *CM* chronic migraine, *NDPH* new daily persistent headache, *PHHT* persistent headache attributed to head trauma

#### Safety and tolerability

Both first time (*n* = 187) and repeat admission data (*n* = 176) were collected and analyzed.

Mean total dosage of DHE given was 10.0 mg, 10.2 mg, and 10.7 mg for patients with CM, NDPH, and PHHT respectively.

Change in mean BARF score throughout the admission from baseline was 0.9 (SD 0.9), 0.6 (SD 0.6), and 0.7 (SD 0.6) for CM, NDPH, and PHHT groups respectively. Among all admissions 85% (308/363) of patients reported nausea.

Side effects other than nausea were reported in 170 (68%) of patients with CM, 39 (63%) of patients with NDPH, and 31 (63%) of patients with PHHT. The most common side effect reported was mild leg cramping. All other side effects were experienced by fewer than 10% of patients (Table 6).

**Table 6:** Reported side effects during first-time and repeated admissions for DHE

Side Effect	Number of patients (%) Total number of admissions = 363
Nausea	308/363 (84.8%)
Mild Leg Cramping	193/363 (53.2%)
Chest Pain	26/363 (7.2%)
Worsening Headache during infusion	26/363 (7.2%)
Severe Leg cramping	9/363 (2.5%)
Deep Vein Thrombosis	3/363 (0.8%)

Deep vein thrombosis was reported in two patients during their first admissions and one patient during a repeat admission. All three patients had peripherally inserted central catheters. Six patients with CM (2.0%) and 1 patient with PHHT (4%) required repeated electrocardiogram for chest tightness. However, no clinically significant cardiac events occurred. No serious adverse events (such as death, congenital anomaly, or major medical events (myocardial infarction/stroke, serotonin syndrome) were reported. Five patients were unable to complete the entire standardized protocol due to side effects (4 patients due to nausea and 1 patient due to deep venous thrombosis).

## Discussion

In children and youth with chronic headache disorders, repetitive IV DHE infusions paired with a new preventive strategy significantly improved medium term outcomes. Patients with chronic migraine had significant decreases in monthly headaches days, baseline headache intensity, number of severe headache days per month, and monthly days of acute medication use. Patients with NDPH had a significant decrease in headache intensity, and patients with NDPH and PHHT had a significant decrease in acute medication usage.

While most patients experienced side effects during their hospitalization, most were mild. A previous study [17] found an increased risk of DVT during DHE administration specifically with administration through central access. In our study population, less than 1% experienced DVT, but the use of central access lines was limited. Previous evidence supports that better nausea control predicts improved outcomes during IV DHE administration [14], which our study did not find. However, our patients adhered to dual antiemetic therapy with an escalation pathway that successfully controlled this side effect (mean BARF score increase was 0.9 points) which may have mitigated the effect.

Our data support previous studies examining DHE infusions in children and youth. Previous pediatric studies evaluated the acute effect of DHE in pediatric patients with primary headache disorders and found it improved pain during hospitalization in 60 to 85% of patients with both acute and chronic headache [13, 23]. Our data add to this by examining the medium-term outcomes of repetitive IV DHE plus preventive treatment adjustment 6–8 weeks after discharge. This is in line with previous adult studies [10, 24].Our data are consistent with that of Srouja et al. [13] that the treatment seems to be generally safe, noting that serious adverse effects were not appreciated. Although in adults severe adverse effects of DHE are reported such as myocardial infarction and arrhythmias, these side effects are generally rare [25, 26]. Furthermore, recent data support its use even in adults with cardiovascular risk factors [27]. Given the need for prolonged hospitalization to complete this standardized protocol, we encourage its use in patients with chronic, refractory headache disorders or patients experiencing significant functional limitations. However, DHE may have varying regulatory recommendations in some countries affecting use [28].

It is important to acknowledge that headache resolution was not achieved in any group; rather, pain intensity was improved. Improvement of pain intensity has been shown to significantly and dramatically improve a patient’s quality of life, especially in the aspects of social function, vitality, and mental health [29, 30]. Katz researched the relationship between four degrees of perceived pain (no pain, mild pain, moderate pain, and severe pain) and measured how each category correlates with quality of life. All metrics studied except for bodily pain drastically improved when a person was able to recodify from moderate pain to mild pain [29]. Furthermore, adolescent patients with continuous headache due to chronic migraine and their parents have self-reported that decreasing days of severe headache was the most valuable outcome of their treatment plan [31]. Accordingly, interventions that reduce pain for patients with headache disorders may have a beneficial effect irrespective of whether complete headache resolution is achieved.

Strengths of this study include that care was provided by an experienced team of fellowship-trained, United Council of Neurologic Subspecialty–certified headache medicine physicians who standardized the care and data collection. Data were also obtained from the medical records in a standardized manner. Overall, the sample size is fairly large for this patient population. Multiple headache disorders were studied, including patients with refractory, continuous headache disorders, who are typically excluded from clinical trials. Multiple metrics to describe headache improvement were investigated, providing a more nuanced view into the possible areas that may be impacted by DHE treatment.

There were several limitations to this study. It was conducted at a single institution with a large child and adolescent headache center, which may limit the generalizability. Additionally, it is a retrospective chart review study, and although a standard chart template was used, some data were missing. This was mitigated by excluding patients with more than 20% missing data. A placebo control group was not available for comparison, so while we cannot exclude a placebo effect or natural history effect, it is less likely given that most patients’ headache disorders had been refractory to multiple previous treatment strategies. Patients were seen by a dedicated headache provider daily during admission, and while there is no formal or standardized education, frequent access to the provider may have impacted the results. We also acknowledge that the concurrent initiation of DHE and a new preventive strategy may limit extrapolation regarding the benefits of solely DHE in children and youth. However, this is the standard of care at our institution due to a possible synergistic effect of DHE paired with a new preventive rather than DHE alone in patients with chronic headache disorders, as reported in the adult population [10].

Given the retrospective nature of our study, these preliminary results require future prospective studies with control groups to validate our findings. Moreover, studies should be conducted evaluating longer term outcomes. While our study specifically evaluated the outcomes of patients who utilized DHE for the first time, studies are needed to understand the utility of subsequent admissions for patients with chronic, refractory headache disorders. Finally, additional studies assessing whether DHE formulations that can be administered non-invasively are useful in this population are indicated if the bioavailability in commercially available options could be optimized [15].

## Conclusion

In children and youth with chronic headache disorders, repetitive infusions of DHE followed by a new or updated preventive strategy improved baseline headache frequency, baseline headache intensity, number of severe headache days, and/or acute medication usage. While side effects were experienced by most patients, they were mild and easily treatable. While this protocol requires admission into a hospital setting for delivery, it could be developed throughout hospital systems in which IV DHE is available as a treatment option for refractory headache disorders. Prospective studies that include a control group are needed to further evaluate the role of repetitive IV DHE administration in this population.

## Supplementary Information

Supplementary Material 1.

## Data Availability

The data that support the findings of this study are available from University of California San Francisco, but restrictions apply to the availability of these data. Data are however available from the authors upon reasonable request and with permission of University of California, San Francisco.

## Abbreviations

DHE: Dihydroergotamine
CM: Chronic Migraine
NDPH: New Daily Persistent Headache
PHHT: Persistent headache attributed to head trauma
IV: Intravenous
CGRP: Calcitonin Gene-Related Peptide
SD: Standard Deviation
CI: Confidence Interval
BARF: Baxter Animated Retching Faces
DVT: Deep Vein Thrombosis
