# Supplementary material for: Tolerability of repetitive dihydroergotamine infusions paired with an adjustment in preventive treatment strategy in chronic headache disorders in children and youth
Source: J Headache Pain. 2025 Apr 30;26(1):93. doi: 10.1186/s10194-025-02035-x (PMC12042534; doi:10.1186/s10194-025-02035-x)
Supplement: Supplementary file 1 — Supplementary Material 1. [file 10194_2025_2035_MOESM1_ESM.pdf]

Figure 1. Histogram depicting pre-admission and post admission outcomes for patients with chronic migraine. A. Baseline headache intensity, B. Monthly severe headache frequency, and C. Frequency of monthly acute medication use

A.

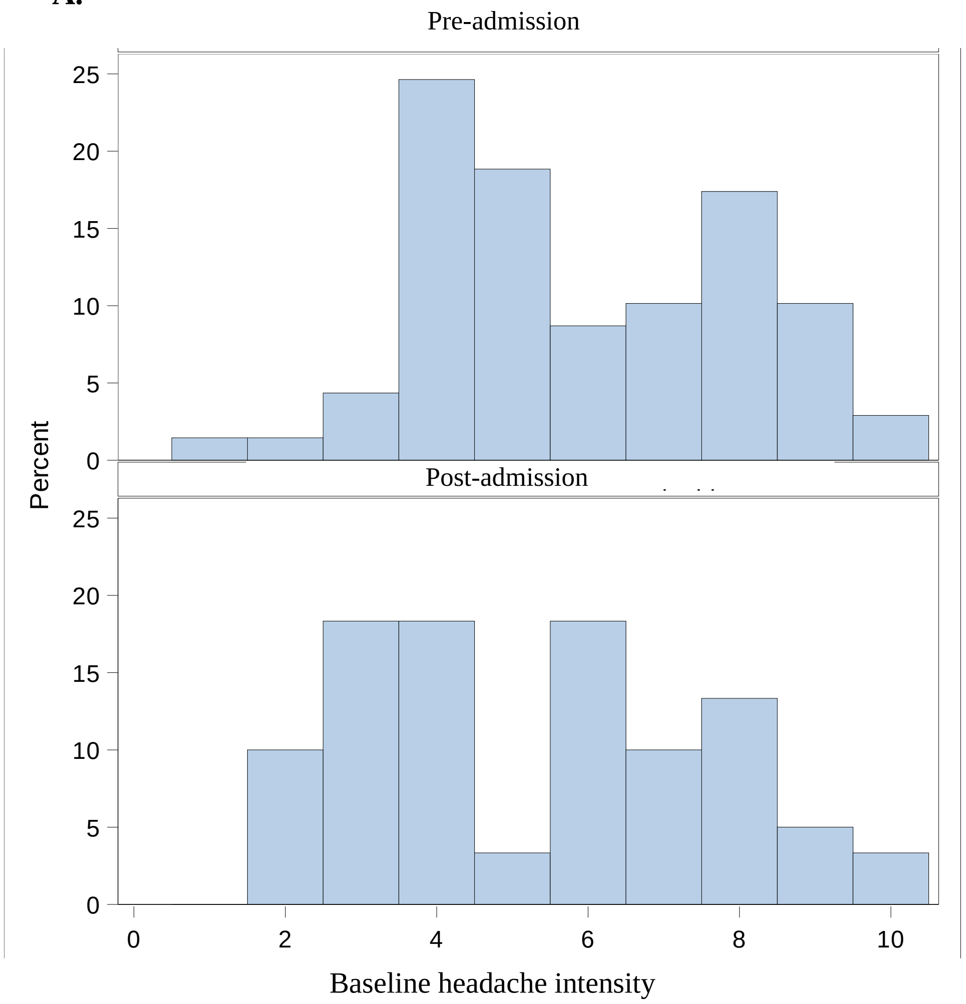

B.

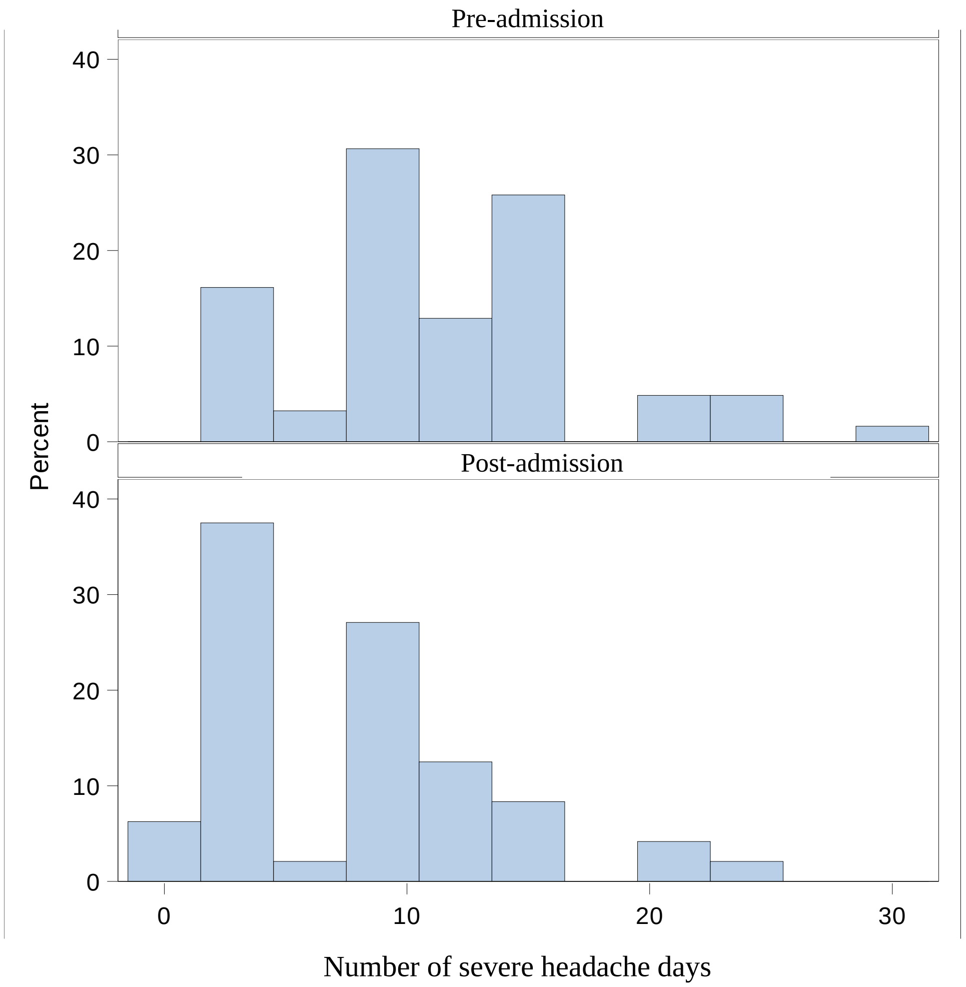
Pre-admission
Post-admission
Percent
0
10
20
30
40
Number of severe headache days

C.

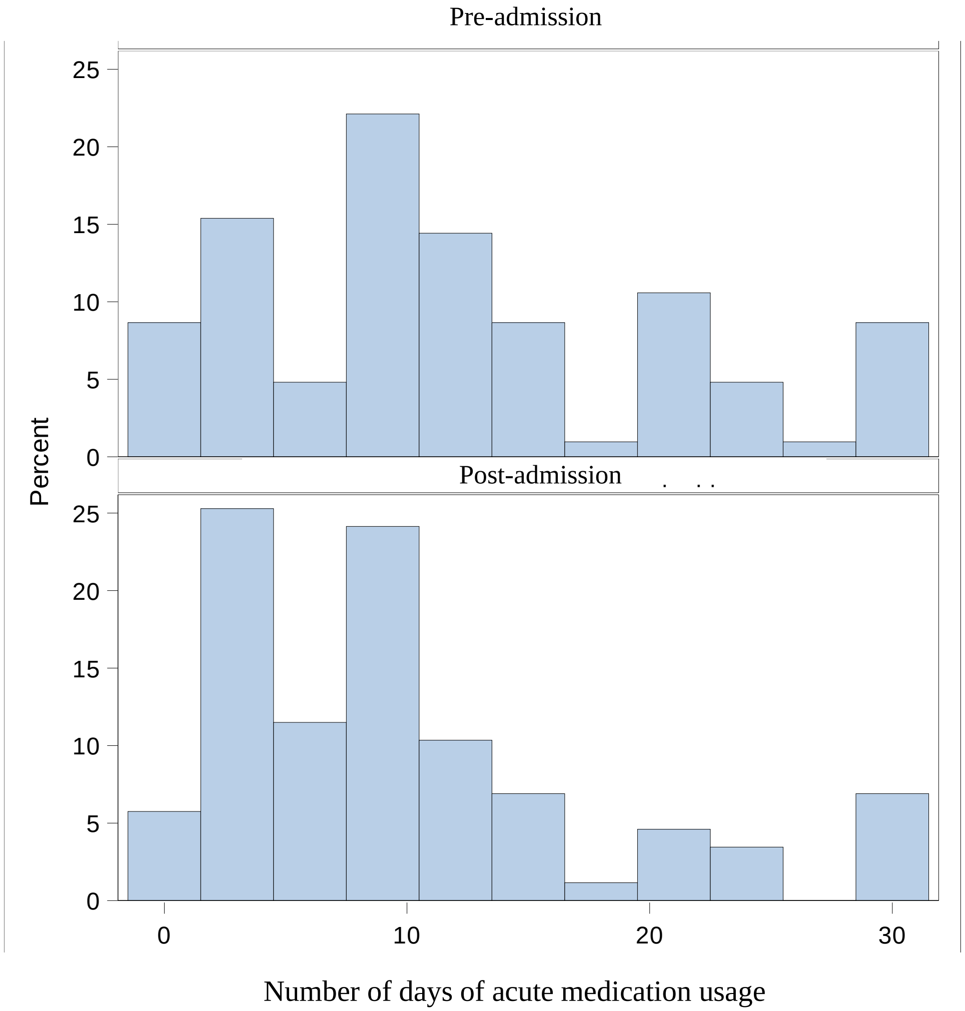
Pre-admission
Post-admission
Percent
0
5
10
15
20
25
0
10
20
30
Number of days of acute medication usage

Figure 2. Histogram depicting pre-admission and post admission outcomes for patients with new daily persistent headache. A. Baseline headache intensity, B. Monthly severe headache frequency, and C. Frequency of monthly acute medication use

A.

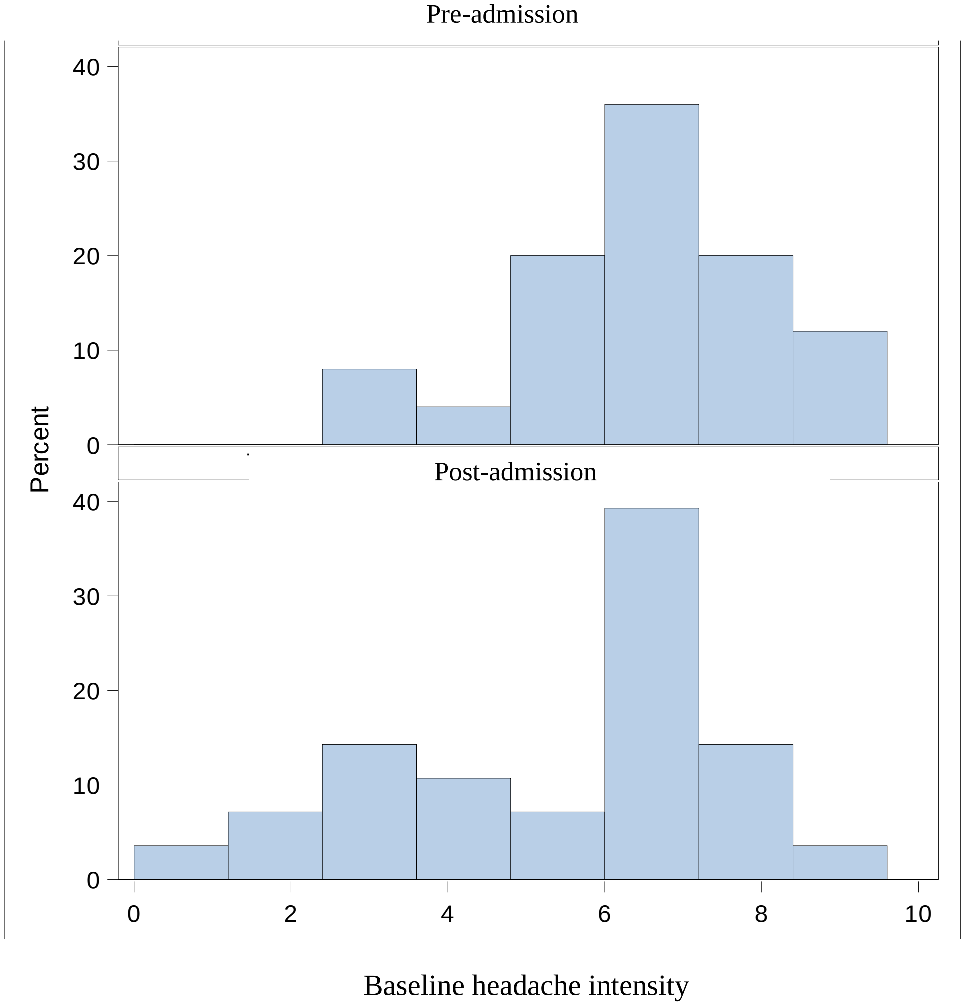

B.

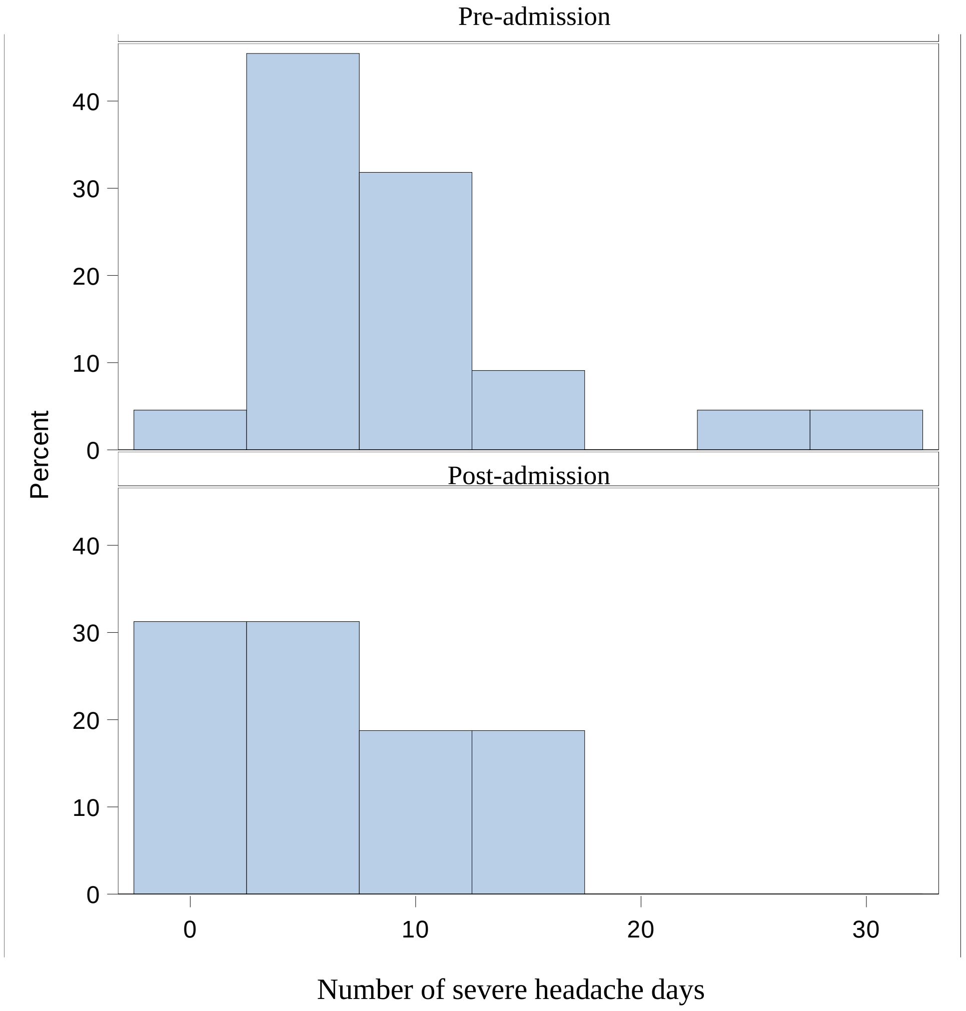
Pre-admission
Post-admission
Percent
40
30
20
10
0
0
10
20
30
Number of severe headache days

C.

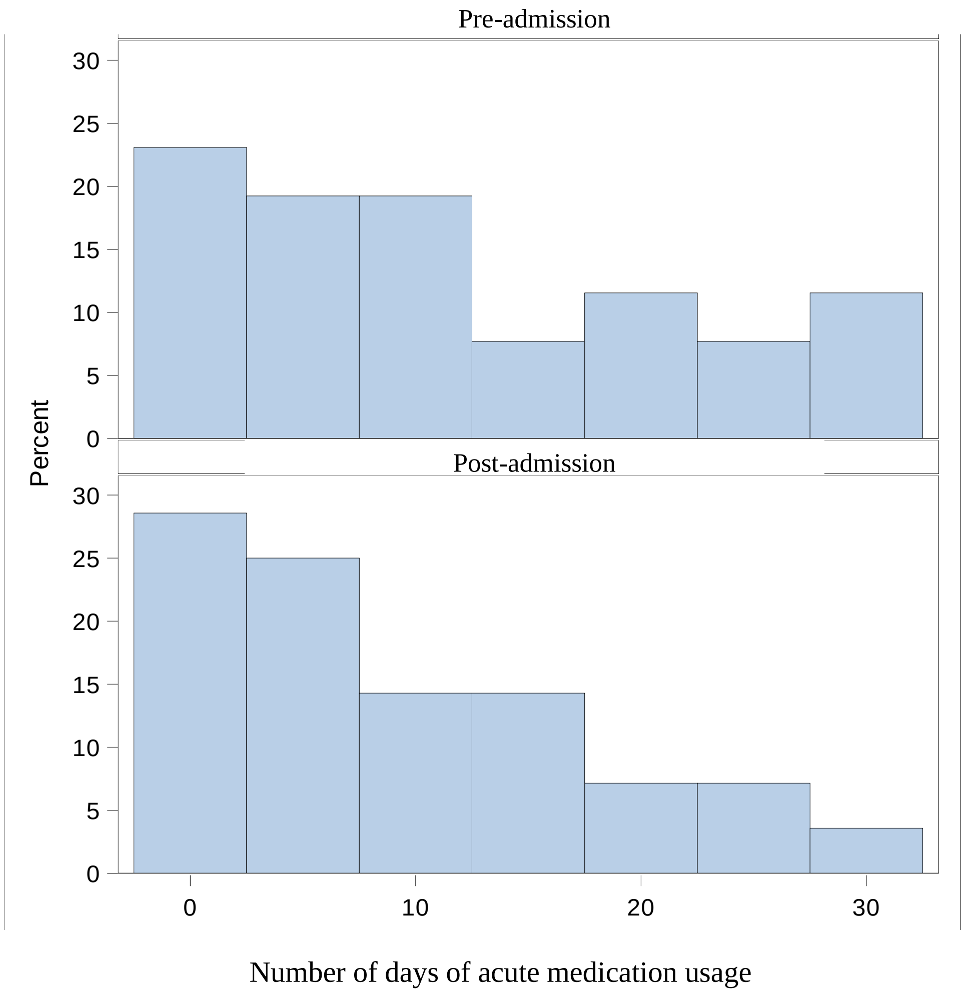
Pre-admission
Post-admission
Percent
0
5
10
15
20
25
30
0
10
20
30
Number of days of acute medication usage

Figure 3. Histogram depicting pre-admission and post admission outcomes for patients with persistent post traumatic headache. A. Baseline headache intensity, B. Monthly severe headache frequency, and C. Frequency of monthly acute medication use

A.

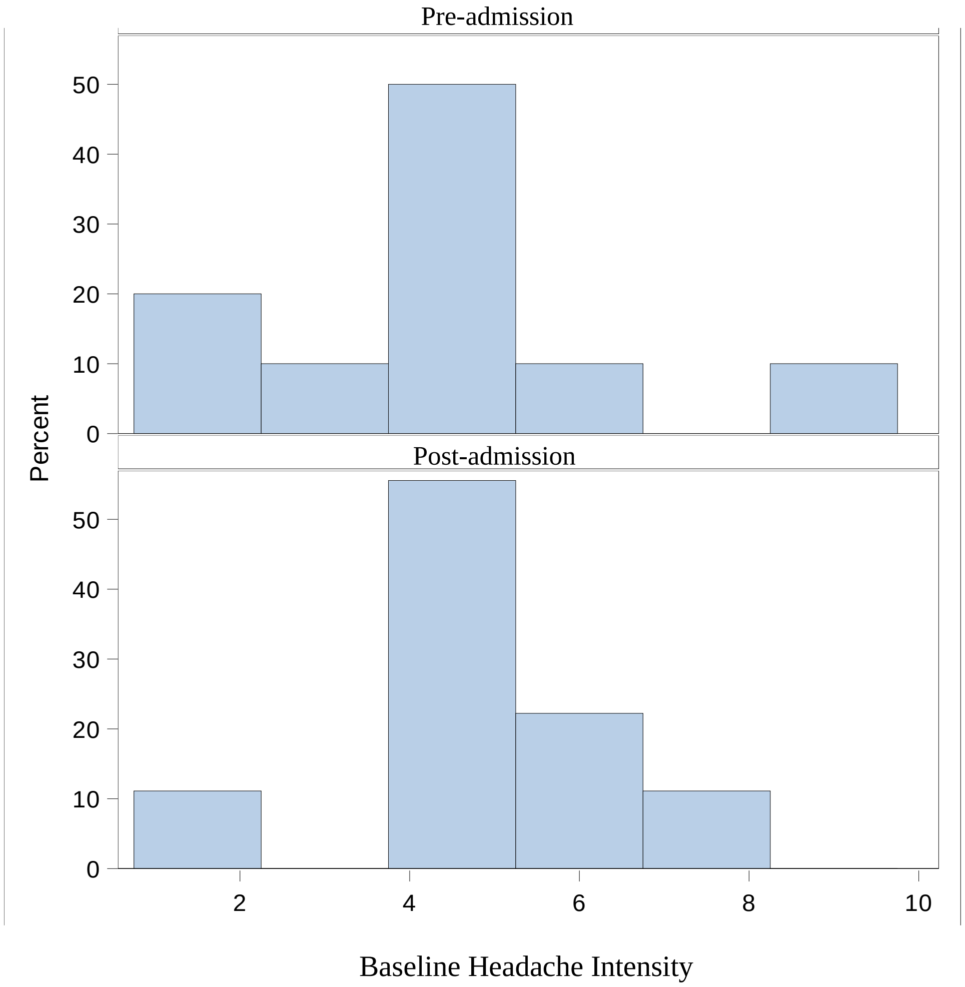

B.

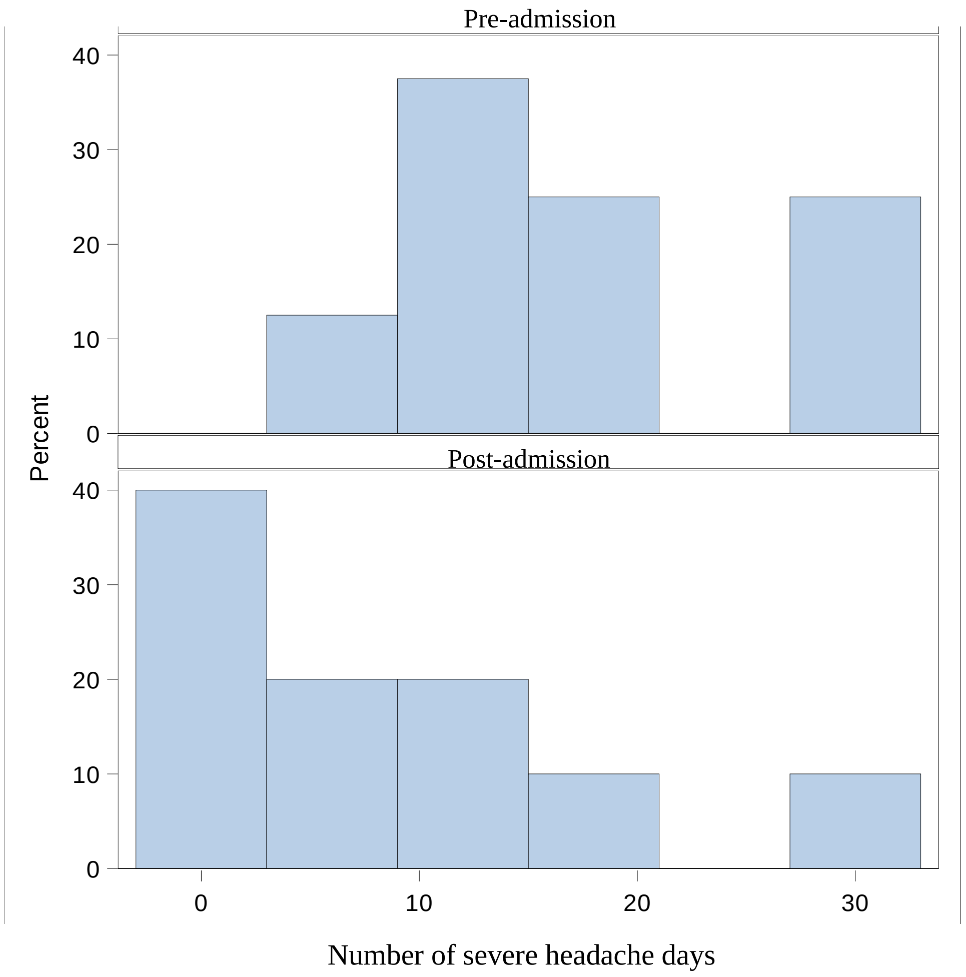
Pre-admission
Post-admission
Percent
0
10
20
30
40
0
10
20
30
40
0
10
20
30
Number of severe headache days

C.

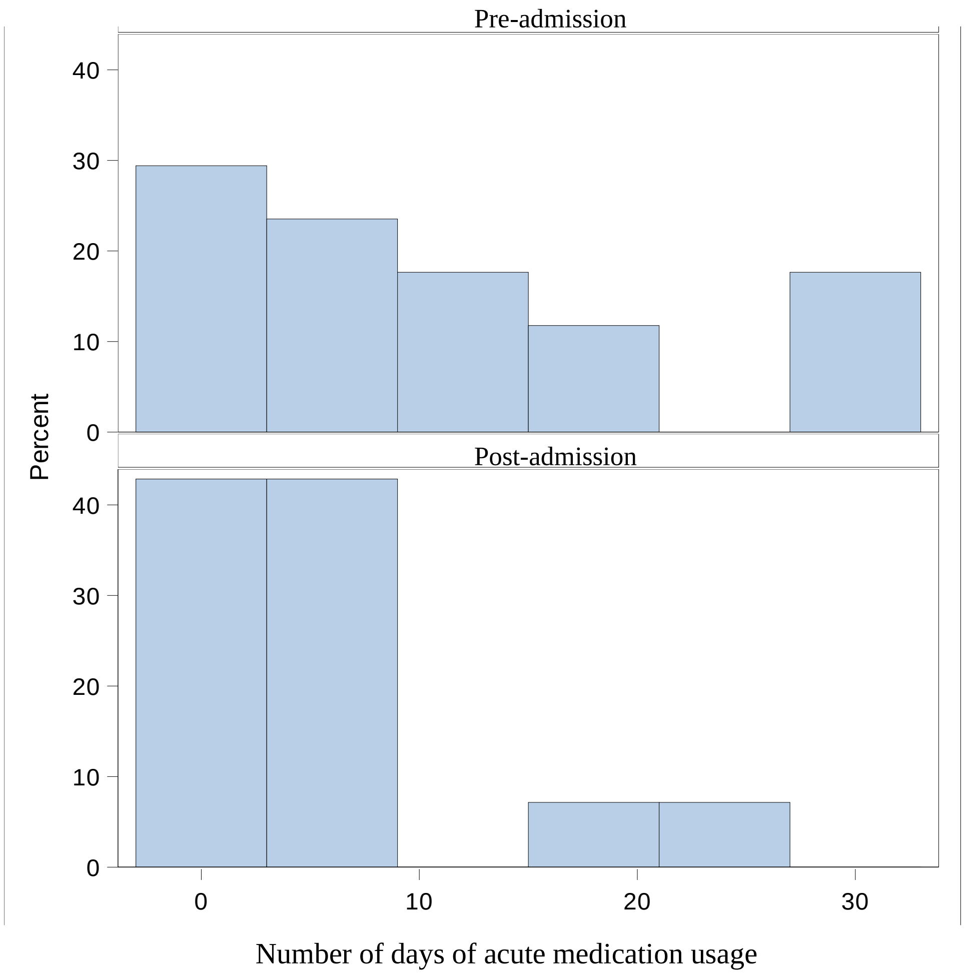
Pre-admission
Post-admission
Percent
0
10
20
30
40
0
10
20
30
Number of days of acute medication usage
